# Reproductive isolation, speciation, and the value of disagreement: A reply to the commentaries on ‘What is reproductive isolation?’

**DOI:** 10.1111/jeb.14082

**Published:** 2022-09-05

**Authors:** Anja M. Westram, Sean Stankowski, Parvathy Surendranadh, Nicholas H. Barton

**Affiliations:** ^1^ Insitute of Science and Technology Austria (ISTA) Klosterneuburg Austria; ^2^ Faculty of Biosciences and Aquaculture Nord University Bodø Norway

## 1 INTRODUCTION

Some say that when the title of a paper is phrased as a question, the authors will not be able to give a clear answer. Our article ‘What is reproductive isolation?’ (Westram et al., 2022a,b) is no exception: noticing that the term ‘Reproductive isolation’ (RI) is frequently used in the literature, yet almost never clearly defined, we tried to come up with a definition—but we quickly ran into numerous complexities that kept us from finding a simple answer. The complexity of the issue is reflected by the commentaries to our article, which highlight the diversity of views among speciation researchers with different interests, different empirical research experiences and different favourite species concepts [a diversity also highlighted by Rosales (2022)]. Some of the commentaries discuss the conceptual (Moyle et al., 2022) and practical (Stuckert & Matute, 2022) limitations of definitions of RI based on gene flow, whereas Planidin et al. (2022) extend the concept to aspects not explicitly covered by us. Rosales (2022) discusses how scientific definitions are developed and what they mean for the community and analyses our work in this context.

In this response, we elaborate on why we think a definition based on gene flow is appropriate if we want to ‘make explicit and further elaborate’ (Rosales, 2022) the already existing concept of RI. In addition, we consider other important axes along which speciation could be quantified that were highlighted by the commentaries and discuss how they relate to RI. Numerous other interesting points brought up by the commentaries can unfortunately not be discussed here for reasons of space.

## 2 DEFINING RI

Scientific terms and concepts need a clear definition (Butlin, 2022; Rosales, 2022). However, it is difficult to find a clear definition for terms that have long been in use, with vague or varying meanings (Harrison, 2012). It may be impossible to find a definition that encompasses all existing usage and that meets the demands of all users (Moyle, 2022).

After some discussion and debate, we arrived at the following verbal definition: ‘RI is a quantitative measure of the effect of genetic differences on gene flow. RI compares the flow of neutral alleles from one population to another population, given a set of genetic differences that reduce gene flow, with the flow expected without any such differences’ (Westram et al., 2022a,b). However, not all the commentators agreed with this proposal. Butlin (2022) suggests that understanding gene flow is an important part of speciation research, but that the term RI should be restricted to short‐term patterns of interbreeding and hybrid fitness. Moyle (2022) also prefers a definition of RI that focuses on interbreeding, but argues that multiple definitions of RI can coexist to reflect the demands of different researchers and species concepts. In contrast, Mallet and Mullen (2022) argue that RI should not be defined as a precise quantity at all and is better treated as a heuristic with a vague meaning. All authors likely have a similar understanding of the processes driving speciation, and the definition of RI might seem like a semantic issue; but beyond semantics, it is surprising and somewhat disconcerting that a defining aspect of the Biological Species Concept (BSC) (which the majority of speciation researchers prefer; Stankowski & Ravinet, 2021) is interpreted differently by researchers studying similar topics.

What approaches could be used to formulate a definition of RI? One approach is to focus on the meaning of the words ‘reproductive’ and ‘isolation’, independent of the history and current usage of the term—a point made by Moyle (2022). Another option would be to stay true to the historical use of the term, orienting on definitions from Mayr or Dobzhansky. One could also define RI based only on its current usage, perhaps informed by the survey we discuss, or RI could be defined based on what can easily be measured, because a definition we cannot apply practically may seem to have little value. All these approaches have merit, but the resulting definitions may differ.

With our definition, we mostly aimed to do justice to the historical and current use of the term. RI already *has* a meaning—but this meaning is so vague that it is open to varying interpretations. Thus, we aimed to distil the key components of this blurred meaning into something specific and logically consistent. Rosales (2022) discusses this approach and describes it as ‘making explicit and further elaborating the conceptual net’. This was a main aim of our article. We do not claim that our definition of RI captures all aspects of the speciation process, lends itself to easy empirical quantification or supports the BSC over other species concepts. We instead claim that our definition gives clearer form to a concept that has already long existed in the literature and that is widely used today.

What are the common aspects of historical and current usage of the term RI? First, historically, RI has been directly connected to gene flow, as evident in the definitions by both by Mayr and Dobzhansky: RI corresponds to a reduction in gene exchange between populations. Second, the survey results show that researchers use RI to refer to reductions in successful interbreeding between populations and/or the restriction of gene flow between populations. Third, the term is strongly connected to the BSC, where RI is the criterion for speciation. In the original sense, the presence/absence of RI determines whether there is a connection between gene pools and thus whether a population pair has speciated. In current usage, RI is more often seen as a quantitative concept reflecting the position of a population pair in the continuous process of speciation. For example, the ‘speciation continuum’ is assumed to reflect the continuum of RI, or one taxon pair may be described as showing ‘stronger reproductive isolation’ than another. A precise definition of RI must connect these different aspects, unless we want to uncouple the term from historical and current use.

The third usage, in the context of the BSC, is especially tricky when formulating a definition. According to the BSC, species are defined by RI, and RI is sometimes used almost synonymously with the degree of speciation. In our opinion, a definition of RI should therefore reflect speciation in terms of the BSC—otherwise, we risk uncoupling the term from its main use. At the same time, crucially, it would be circular to define RI by reference to the BSC—it must have an independent definition.

We argue that a definition based on gene flow is largely consistent with historical and current use, including usage in relation to the BSC. First, a definition based on gene flow does justice to historical use as it relates to Mayr's (1959) point that RI prevents ‘…pollution by other gene pools’ and is especially closely related to Dobzhansky's (1951) view, where ‘…gene exchange between species is restricted or suppressed owing to genotypically conditioned differences between their populations’. Second, our definition is also compatible with the view of the 47% of researchers in the survey who referred to gene flow or gene exchange in their definition of RI. It is compatible with the organismal view of RI, because reduced interbreeding is the necessary cause of reduction in gene flow. Third, our definition also fulfils the criterion of describing the ‘degree of speciation’ in terms of the BSC—the reduction in gene flow between genetically divergent populations is one appropriate measure of the degree of speciation. Nevertheless, RI (as we define it) is not defined with reference to speciation or the BSC—it has an independent meaning as a measure of the effect of genetic divergence on gene flow, a crucial evolutionary process. Using RI according to our definition therefore does not necessarily imply adherence to the BSC, and the term could be used outside a speciation context. Instead, it reflects a concept essential to understanding the flow of both neutral and selected variation in general.

Moyle (2022) and Butlin (2022) prefer a definition more in the ‘organismal’ direction, based on the generation and fitness of hybrids. Moyle (2022) suggests that this focus more clearly reflects the mechanisms of speciation. Butlin (2022) suggests using ‘isolation’ to encompass both geographical isolation and ‘reproductive isolation’, which he restricts to the short‐term reduction in the generation and fitness of early‐generation hybrids. As discussed above, there are different ways of formulating a definition that are valid. The definitions favoured by Butlin (2022) and Moyle (2022) have the advantage that they may seem closer to the intuitive meaning of the term ‘reproductive’, as they directly relate to the measurement of mating and hybridization. In addition, as Butlin (2022) and Moyle (2022) argue, their perspective allows a clearer path towards feasible experimental approaches. This is an important point—we agree that a definition of RI that does not lend itself to empirical measurement is dissatisfying. Nevertheless, it is also important to note that a definition based on gene flow does not constrain the methods used to quantify RI. For example, in a 2‐deme scenario, we showed that RI for a neutral locus that is unlinked to selected loci can be calculated from the fitness of immigrants, F_1_ hybrids, and backcrosses—and this calculation can be based on existing non‐genetic methods typically associated with organismal thinking. However, our definition also opens the door to methods that use genomic data to estimate RI [as discussed by Stuckert and Matute (2022)], as well as new approaches that combine organismal with genomic data.

Despite being attractive for practical reasons, definitions focused on interbreeding are further removed from the historical definitions of RI by Dobzhansky et al. than those based on gene flow. However, to us, the main issue with organismal definitions of RI—and organismal methods as they are usually applied—is that they will only provide a measure of genetic connectivity under limited circumstances. For example, even if early‐generation hybrids between two parental populations have very low fitness, genetic coherence between these populations may nevertheless be high if fitness is restored to high levels in later generations. Definitions focused on interbreeding can therefore neither generally reflect speciation in terms of the BSC nor are they suitable for understanding the flow of neutral and selected alleles (an important function independent of speciation and species concepts, as discussed above). As highlighted by Rosales (2022), definitions of important scientific concepts influence the course of research. Thus, we disagree with the terminology suggested by Butlin (2022) and prefer a definition based on gene flow for the term RI, but we do agree that both aspects discussed by Butlin (2022; gene flow and short‐term interbreeding) need to be studied (and should have their own, separate terminology), as we discuss below under ‘Axes of speciation’.

A somewhat separate point is that both Mallet and Mullen (2022), and Butlin (2022), suggest that RI (or, in Butlin's terminology, the barrier to gene flow) should be defined also for selected, not just for neutral loci. We have two objections. First, we disagree with Mallet and Mullen's (2022) perspective that considering selected loci is necessary because species could be defined as ‘genotypic clusters’. Although it may seem plausible to define species simply through sets of alleles that are kept together at high frequency [as, e.g., in the *Anopheles* example given by Mallet and Mullen (2022)], this could lead to the fragmentation of populations into innumerable clusters, each defined by different sets of selected loci. In principle, balancing selection could maintain very many polymorphisms, with varying degrees of linkage disequilibrium, in which case definition of a ‘species’ becomes arbitrary. This objection is to the species concept, but regardless of how we define species, a second objection is that defining RI for selected loci confounds gene flow with selection. RI has been, and is, closely associated with gene flow, and this connection is lost if we use it to refer simply to divergence maintained by selection. Effective migration rates measure gene flow at both selected and neutral loci, but to measure the reduction in gene flow caused by genetic differences, we must focus on loci other than the causal loci themselves (i.e. on neutral loci). We find it unhelpful to blur together selection and gene flow, which are distinct aspects of the evolutionary process.

## 3 WHY QUANTIFY SPECIATION?

Given that some vague understanding of RI already exists, and that there is so much disagreement about the exact definition, do we need a quantitative definition at all? Some of the commentaries suggest that a vague (Mallet & Mullen, 2022) or context‐dependent (Moyle, 2022) definition of RI may be preferable. Mallet and Mullen (2022) argue that quantifying the overall reduction in gene flow is difficult, and therefore, a focus on its components—for example the component of assortative mating—may be more useful. Similarly, Butlin (2022) argues that focusing on pre‐and post‐recombination components separately may be more practical, and suggests splitting ‘reproductive isolation’ into various components, including mating patterns and F_1_ viability.

We agree that measuring components separately may be easier in practice and is certainly a valuable research programme. However, we suggest that RI with a quantifiable, gene flow‐based definition fulfils an important function in speciation research: *it measures the degree of speciation in terms of the BSC*. By this, we mean that it can be used to quantify speciation along a one‐dimensional axis—a system can move in both directions along this axis, reflecting the degree of speciation.

As we discuss below, there may be multiple such axes of speciation, and RI as defined by us is only one of them; nevertheless, our definition allows, at least in principle, for a (partial) quantification of speciation. We believe the quantifiability of speciation is crucial: Can we really study speciation if we make no attempt to even roughly quantify it? To take up the temperature analogy discussed by Rosales (2022): if we want to study how certain processes or mechanisms influence temperature, we need to understand and define temperature as something that, at least in principle, can be expressed quantitatively. If we want to know how different factors influence speciation, we need to be able to quantify its extent. For example, in a comparative framework, we can only make comparisons between different taxon pairs if we have some measure of the degree of speciation in each of them. Similarly, we can only understand the contribution of certain barriers to speciation if we can somehow quantify speciation. For example, with our definition, one could measure RI between two diverging populations, and also analyse local adaptation in transplant experiments. Then, one could ask: How much of the RI is generated by local adaptation?, thereby addressing the relative contribution of local adaptation to speciation.

However, importantly, RI (in our definition) quantifies *speciation only along a single axis ‐ the one that is central to the BSC*. There are multiple axes along which speciation could be quantified. We argue that some of the disagreements in the different commentaries reflect preferences for different axes—all these axes are real, but different researchers focus on different axes and might even label different ones as ‘RI’. We believe that the main contribution of this discussion to our field is indeed not the determination of one unified definition of RI, but an increased awareness that RI is a subtle concept, and that these different axes exist, beyond RI. We can then disentangle them and ask how we can measure a system's position on each of them.

In the following, we discuss different points from the commentaries that involve different axes, under the premise that a quantitative view of speciation is useful and essential for our field. We first discuss different axes of speciation in general—explaining why RI alone cannot reflect all aspects of speciation. We then specifically discuss several different axes relating to gene flow.

## 4 AXES OF SPECIATION

Reproductive isolation, as we defined it, reflects one ‘axis’ of speciation in the sense that movement along this axis means a change in the degree of biological speciation, through the modulation of gene flow. However, speciation is a multidimensional process, involving evolution along multiple ‘axes’, each corresponding to a different aspect of what a species is: for example morphological divergence, ecological divergence and untangling of genealogies. This means that, even in simple cases where RI can be described by a single number, this measure by no means captures all the relevant factors. One can think of different species concepts as corresponding to different such axes, where, for example, RI measures the reduction in gene flow, corresponding to the BSC, whereas morphological divergence reflects the morphological species concept. The extent of clustering at selected loci, discussed by Mallet and Mullen (2022), could be considered another axis. Of course, movement along the different axes may be associated—for example, ecological niche divergence may include morphological change, and also correspond to a genetically‐based reduction in gene flow. A key issue in speciation research is to understand how these different aspects of speciation interact with each other.

Nevertheless, considering different axes independently is important to understand speciation as a whole. A particularly powerful example is the role of RI vs niche divergence, by which populations evolve to use different limiting resources. Movement of a system along these two axes need not always be correlated—for example, RI could be strong despite little niche divergence if driven by intrinsic incompatibilities. RI alone cannot ensure the long‐term persistence of a new species: acting alone, it would only separate a population into ever smaller fragments. In the long term, species must also find a distinct ecological niche, which allows them to coexist with other species. Thus, as mentioned by Stuckert and Matute (2022), RI does not necessarily determine speciation rates at the macroevolutionary scale. In this sense, both RI and ecological divergence are required for the evolution of stable separate species—neither the RI axis nor the niche divergence axis alone fully describes speciation.

It is also important to note, as pointed out by Moyle (2022) and Butlin (2022), that under our definition, RI describes an *outcome* (the reduction in gene flow), but not the particular underlying causes, and therefore includes the effects of different types of barriers (e.g. mating patterns and intrinsic incompatibilities). These are not themselves different axes of speciation, as they each influence speciation by altering RI (and maybe also affecting other axes), and so we refer to them as ‘components’ of RI (i.e. our use of ‘components’ is related to that of Butlin, 2022). It is certainly important to understand these components separately, for example by measuring the contributions of intrinsic and extrinsic, and of pre‐and postzygotic barriers, in the traditional way (Coyne & Orr, 2004; Sobel & Chen, 2014). Butlin (2022) makes the point that it is important to analyse how gene flow and organismal measures are related, and we agree. In fact, without a quantitative measure of RI, we cannot compare the relative importance of different components of RI. The scarcity of work connecting these different levels is a key gap in speciation research, and studies that ask about the effect on gene flow of apparent barriers at the organismal level are crucial (e.g. Irwin, 2020; Perini et al., 2020 for assortative mating). Similarly, multiple processes contribute to the establishment of RI, including local adaptation, snowballing of intrinsic incompatibilities and reinforcement. RI, defined as a reduction in gene flow, summarizes the outcome of all of these together. Ideally, we need to relate each individual process to RI by asking how much it contributes to current RI and thus facilitates (biological) speciation.

## 5 AXES RELATED TO GENE FLOW

We argue that RI does not capture all aspects of gene flow relevant to speciation—there are multiple axes of speciation that relate to a reduction in gene flow. We highlight some of these axes specifically, because they are closely related to RI and are reflected in the commentaries. One example is highlighted by Butlin (2022), who argues that geographical and ‘reproductive’ barriers cannot clearly be separated. For example, if a geographical barrier can hardly be crossed by the individuals of the two separated populations, this separation still has a genetic component (as the populations could in principle have evolved traits that enable them to cross the barrier). While Butlin (2022) combines anything that has a genetic *basis* (which can be the same in both diverging populations) into ‘isolation’, our definition of RI, in contrast, focuses only on reductions in gene flow caused by genetic *differences* between populations. This directly relates to the distinction between one‐ and two‐allele mechanisms made by Felsenstein (1981). Butlin's (2022) perspective has the advantage that it includes effects of, for example, reinforcement favouring the same allele in both diverging populations, whereas our approach would ignore the effects of such alleles even though they clearly evolve as part of the speciation process. On the other hand, Butlin's (2022) perspective has the disadvantage that it mixes one‐ and two‐allele effects. We thus propose that reductions in gene flow due to genetic differences and reductions in gene flow due to shared genetic changes simply can be viewed as different axes of speciation.

A similar point applies to the role of epigenetic effects, discussed by Planidin et al. (2022). The concept of reproductive isolation was developed before it was known that genetic information is encoded in DNA sequence and can easily be transferred to other mechanisms of inheritance. As Planidin et al. (2022) show, epigenetic inheritance based on DNA methylation can be incorporated into our measure, using essentially the same mathematical analysis. Similar arguments apply to other forms of heritability, for example cultural differences (e.g. song dialects, e.g. in *Zonotrichia*; Slabbekoorn & Smith, 2002), *Wolbachia* infection (Werren, 1998) or environmental effects of the population of origin on the compatibility of migrants with their new population. One could argue that the reduction in gene flow due to these kinds of heritable differences constitutes another axis of speciation separate from RI, perhaps on the grounds that they tend to be less stable than genetic differences. On the other hand, one may prefer to keep a simpler definition of RI that does not introduce extra criteria regarding the form of inheritance or stability of the barrier to gene flow.

Other axes might be less obvious. According to our definition, RI relates to the reduction in gene flow due to genetic differences between focal populations. However, when focusing on spatially separate populations, this view implies that RI depends on the individuals *between* the focal populations as well, whose genetic composition will affect the realized gene flow between the focal populations. It is possible that populations that are well‐connected via compatible genotypes in nature are incompatible when directly crossed in the laboratory—indeed, this is likely, since fit intermediate genotypes will be selected, and there are many such examples (e.g. in shrews and grasshoppers; Hatfield et al., 1992; Virdee & Hewitt, 1994). The issue is illustrated even more clearly by cases of ‘ring species’, where neighbouring populations are reproductively isolated when in direct contact, but connected via a ‘ring’ of compatible genotypes that allows for gene flow (Irwin et al., 2001). These scenarios would generate low RI under our definition, as the level of realized gene flow between populations is high, and in that sense, the degree of speciation is low. However, one could argue that there is another axis of speciation that instead reflects compatibility between the focal populations when individuals are placed together (either artificially in the laboratory for those cases connected by fit intermediate genotypes, or naturally in ring species). This view is justified because the accumulation of incompatibilities between the focal populations certainly reflects an increase in the degree of speciation in some sense—for example, many researchers would agree that incompatibility in a laboratory setting indicates the presence of separate species. We thus argue that RI between two populations in the natural context and the reduction in gene flow between directly confronted genotypes represent different axes of speciation.

We believe that it is important to disentangle these different axes of speciation, all related to gene flow, but distinct from RI as we define it and as traditionally used. This will lead to a clearer view of the speciation process.

Apart from this general complexity of the speciation process, we strongly agree with the commentaries highlighting that the axis represented by RI (as defined by us) itself is complex, difficult to quantify and hard to compare between systems—this was, in fact, one of the key points that we strove to make in our original article. Even if we define RI based on the reduction in gene flow due to genetic differences, this definition is only straightforward in very simple cases (e.g. two demes at equilibrium). In those cases, RI can potentially be described by a single number (either for an unlinked neutral locus or at a specific position on the genome). In more complex spatial or temporal settings, there are multiple possible values of RI that reflect gene flow between different possible spatial groups and for different possible timespans. This complexity is criticized by Moyle (2022), as it can preclude comparisons across systems. We agree that this is problematic—but it is a result of the actual complexity of nature, not of a bad choice of definition. Despite its complexity, abandoning attempts to define RI as a quantity would mean abandoning opportunities to deepen our understanding. We believe that, just as it is important to identify different axes of speciation, it is also important to identify different components and context‐dependencies of RI, to quantify them separately and to understand their relationships.

## 6 CONCLUSIONS

To some extent, the search for a definition of RI is semantics. The framework suggested by Butlin (2022), for example, does not differ from ours in its understanding of mechanism, as far as we can see. As for other commonly used, but ill‐defined terms in evolutionary biology (effective population size, diversity, species, …), the community may never agree to use a single definition. We thus recommend that authors at least briefly describe how they define the term RI when they use it in a paper or talk, in order to avoid misunderstandings.

Quantitative thinking about speciation is necessary to understand and compare different aspects of the process within and between systems—and yet, speciation can usually not be encapsulated by a single number. What, then, are we to do? First, we suggest that, within the framework of the BSC, we need to deliberately study simple systems (e.g. small sets of a few islands) both empirically and theoretically, to better understand how we can quantify gene flow and how RI varies cross timescales. Second, we suggest that we need to embrace the complexity of speciation and appreciate and separate its different axes more clearly—independent of how we label them. For example, the evolution of genetic differences that reduce gene flow, the evolution of one‐allele mechanisms that reduce gene flow, and the evolution of niche divergence, allowing for coexistence, may all contribute to speciation in their own way—but their contributions are not measured on the same scale. There is already a large body of work quantifying the position of population pairs on different axes—but this work is often done without an appreciation of the presence of other axes. We need to understand how movement along different axes is related, and how positive feedback between different axes could facilitate the evolution of separate species. This perspective requires a synthesis between the views of researchers who study speciation from diverse angles. Some of the disagreements among the present set of articles may simply point us to exactly those aspects of speciation where more synthesis and a broader view of speciation are needed. We thus hope that the discussion captured in this set of papers is valuable despite, and indeed because of, the presence of disagreement.

## AUTHOR CONTRIBUTIONS

**Anja M. Westram:** Conceptualization (equal); writing – original draft (lead); writing – review and editing (equal). **Sean Stankowski:** Conceptualization (equal); writing – review and editing (equal). **Parvathy Surendranadh:** Conceptualization (equal); writing – review and editing (equal). **Nick Barton:** Conceptualization (equal); writing – review and editing (equal).

## CONFLICT OF INTEREST

The authors declare no conflict of interest.

### PEER REVIEW

The peer review history for this article is available at https://publons.com/publon/10.1111/jeb.14082.

## Data Availability

No new data were used in this manuscript.
